# Farming of Plant-Based Veterinary Vaccines and Their Applications for Disease Prevention in Animals

**DOI:** 10.1155/2015/936940

**Published:** 2015-08-13

**Authors:** Pit Sze Liew, Mohd Hair-Bejo

**Affiliations:** Department of Veterinary Pathology and Microbiology, Faculty of Veterinary Medicine, Universiti Putra Malaysia, 43400 Serdang, Malaysia

## Abstract

Plants have been studied for the production of pharmaceutical compounds for more than two decades now. Ever since the plant-made poultry vaccine against Newcastle disease virus made a breakthrough and went all the way to obtain regulatory approval, research to use plants for expression and delivery of vaccine proteins for animals was intensified. Indeed, in view of the high production costs of veterinary vaccines, plants represent attractive biofactories and offer many promising advantages in the production of recombinant vaccine proteins. Furthermore, the possibility of conducting immunogenicity and challenge studies in target animals has greatly exaggerated the progress. Although there are no edible plant-produced animal vaccines in the market, plant-based vaccine technology has great potentials. In this review, development, uses, and advantages of plant-based recombinant protein production in various expression platforms are discussed. In addition, examples of plant-based veterinary vaccines showing strong indication in terms of efficacy in animal disease prevention are also described.

## 1. Introduction

Plant molecular farming is a term used to describe the application of molecular biological techniques to the synthesis of commercial products in plants, which include a variety of carbohydrates, fats, and proteins, as well as secondary products [1]. The process of manufacturing a plant-based vaccine in the plant green factory begins with the selection of a target antigen of interest. The vaccine candidate is cloned into a plant expression cassette that is capable of promoting and terminating transgene expression. The expression cassette is then delivered into a plant for the production of a recombinant protein [2].

The delivery of an expression cassette carrying the gene of interest into the plant cells could be achieved by either stable or transient transformation. Transient gene expression represents a rapid and convenient system for verification and characterisation of the target gene product. Notwithstanding, it is now a routine practice in molecular farming for the production of foreign proteins [3]. The process circumvents the long development time and low protein accumulation levels associated with the use of transgenic plants. Besides, as the foreign protein production is only temporary, it requires no selection method to identify transformed plant cells. Compared to transient expression systems, the major advantage of stable transgenic lines is that the candidate gene sequence is incorporated into the plant genome and thus the acquired protein production trait is inherited. This allows the transfer of the desired character to the next and over multiple generations [4]. Thus, seed stock could be established, which assures the continuing availability of the stock [5].

## 2. Brief History of Development of Plant-Based Recombinant Protein Production

The idea of plant molecular farming was burgeoning about 26 years ago when the proof of concept recombinant plant-derived pharmaceutical proteins was reported (Table 1; [1, 6]). Among these, biologically active human interferon alpha D was produced in turnip following inoculation with mutant cauliflower mosaic virus carrying human interferon alpha D at its ORF II [7]. In the same year, tobacco plants expressing either gamma or kappa immunoglobulin chains of mouse were crossed to generate progeny expressing both chains of immunoglobulin, showing the ability of plants to assemble heterologous biomolecules [8]. Then, the production of human serum albumin in tobacco and potato plants which was identical to the authentic human protein was reported [9].

The idea has since expanded to the production of many industrial and agricultural recombinant enzymes. The leading examples were avidin [12], *β*-glucuronidase [13], and trypsin [14]. Besides, the use of plants for the production of foreign proteins especially biomedically important materials is wide and varied. Several biopharmaceuticals like growth hormones, human blood components, and cytokines have been expressed in plants [6, 11]. Furthermore, many medically related proteins including antibodies or vaccines for human and veterinary use are continually being explored and developed. The monoclonal antibody against hepatitis B surface antigen (HBsAg), expressed in tobacco, was the first commercialized plant-derived antibody. Being marketed in Cuba, it has replaced the mouse derived monoclonal antibody for the routine purification of recombinant HBsAg for hepatitis B vaccine production [10]. Plant-made antibodies currently in the pipeline for commercialization include those against* Streptococcus mutans* and non-Hodgkin's lymphoma [6, 11]. The monoclonal secretory antibody against* S. mutans* made in plant was shown to prevent colonisation of microbes in the human oral cavity [22]. It has since been approved as a medical device in 2003 by the European regulatory authority for human use and commercialized as CaroRx [6].

On the other hand, a variety of vaccine antigens have been well tested for expression in plants, be it for human or veterinary applications. Among the vaccine targets intended for human use,* S. mutans *surface protein A for dental caries and HBsAg for viral hepatitis B were the first to be explored [23]. It has been demonstrated that the plant-made 22 nm particles of HBsAg were similar to HBsAg particles derived from human serum and recombinant yeast, both antigenically and physically [15]. Subsequently, various vaccine candidates including* E. coli *heat-labile enterotoxin B subunit (LT-B), Norwalk virus particles, and cholera toxin B subunit have been expressed in plants [23, 24]. The bacterial antigen LT-B made the first proof of concept for edible plant-produced vaccines by showing that the orally delivered vaccine conferred protective efficacy to mice [18]. Moreover, the vaccine targeting antigens like viral hepatitis B, LT-B, and Norwalk virus are heading towards advanced stages of development and have completed the phase I clinical trials [6]. Notwithstanding, many vaccine antigens are making their way forward although they were in the early stage of development. These include and are not limited to vaccines for rotavirus infection [25], measles [26, 27], human immunodeficiency virus type 1 [28–30], human cytomegalovirus [31], respiratory syncytial virus [32, 33],* Staphylococcus aureus* [34],* Pseudomonas aeruginosa* [35, 36], and* Plasmodium falciparum* [37].

However, it was a plant-made poultry vaccine against Newcastle disease virus (NDV) that turned out to be the first plant-based vaccine to obtain regulatory approval from the United States Department of Agriculture, Center for Veterinary Biologics [21]. The NDV vaccine was shown to confer more than 90% protection rate in chicken upon challenge with NDV. Although the proof of concept poultry vaccine has not been commercialized and there are no other veterinary vaccines introduced into the market since then, animal pathogens have become the focus for expression in plants [38]. Indeed, the number and range of candidate antigens from animal microbes and viruses that have been expressed in plants are extensive [39]. The possibility of carrying out challenge experiments in specific animal species of interest has encouraged and resulted in enthusiastic development of plant-made veterinary vaccines.

## 3. Merits of Plant Production System

Since the early 1990s, plants have gained an additional role of being bioreactors in molecular farming for new drugs and vaccines [38]. Whole plants, either by stable or transient transformation, were used to produce foreign target antigens of interest [40] that have the potential of being applied in routine vaccination strategies. Plants, therefore, represent attractive alternatives for vaccine production. Different parts of plants like the leaf and stem tissues, seeds, and fruits and root vegetables have been used for production of foreign proteins. Some of the commonest species of plants used include small flowering weed* Arabidopsis thaliana* that is widely used in plant science, tobaccos, alfalfa, spinach, potatoes, rice, beans, maize, tomatoes, strawberries, carrots, and many more [3]. Initially conceptualized as a platform for production of edible vaccines [41], expression of foreign proteins in plants aims to reduce the use of needles and cold chain for vaccine delivery especially in the developing countries. Besides, should the food plants be used for foreign protein expression especially for vaccines, they could be consumed directly or with only minimal processing. Nevertheless, plants offer many other advantages over other production systems.

The production cost in plants is only a fraction of that of mammalian cell systems and between 10 and 50 times lower than microbial system like* E. coli* fermentation for producing the same protein [42, 43]. The farming of transgenic plants requires relatively basic and economical propagation materials like sunlight, water, and nutrients. Furthermore, the harvest and downstream processing of transgenic plants require an uncomplicated technology and the scale-up of production is simple and rapid as they can be done by increasing the cultivation area [41].

Production of foreign proteins in plants is generally considered safe when compared to mammalian cell systems, as they are less likely to harbour microbes or prions that are pathogenic to humans or animals [41]. The conventional live veterinary vaccines especially of viral origins, intended for use in poultry, are typically propagated in chicken embryos or cell culture systems. These vaccines consist mainly of attenuated virus strains that have lost their virulent properties but are still replicative and demonstrate the desired antigenic features. Although the vaccines can mimic the course of naturally acquired immunity, they bear the risk of reversion to virulence, which could result in infection instead of protection [44]. Moreover, chicken embryos or animal cells propagation systems carry the inherent threat for unintentional contamination because they are rich in nutrients and thus susceptible to contamination. The avian leukosis virus has been found as a contaminant in commercial Marek's disease vaccines of poultry [45]. Although the contaminated vaccines were promptly removed from the market, it was reported that routine quality assurance measures had failed to detect the contaminant virus. This has undeniably raised safety issues of vaccine production in animal-derived sources. Plants, when used as production system, have lower risks of contamination by extraneous infectious agents.

Other than the apparent advantages in terms of cost, scalability, and safety, plant expression platforms are able to carry out posttranslational modification of proteins like disulfide bonds formation and glycosylation [46]. The proteins could be targeted to and retained in the endoplasmic reticulum of the cell to allow N-glycosylation and avoid complex-type N-glycan modifications by the Golgi apparatus [43]. Being more structurally closer to those of insect cell expression system, the use of plant system, however, would require modification and harmonization should species-specific N-glycosylation be needed to produce therapeutic glycoproteins for veterinary or human purposes [47, 48]. While animal cell cultures are able to carry out posttranslational modification and reproduce glycoproteins with N-glycan structures specific to the animal species they are derived from, they are compensated by the apparent high production cost [49]. As with the insect cell and plant expression systems, the use of mammalian cell cultures from nonhuman origin for expression of glycoproteins for human use would need to be humanized [49]. Similarly, although the microbial production systems like* E. coli* and yeasts are relatively much cheaper than mammalian cell systems, they are not be able to synthesize glycosylated proteins with desired biological properties [48]. As bacteria do not glycosylate, while yeasts may hyperglycosylate, the immunogenicity of the proteins produced might be affected [46].

## 4. Plant-Based Expression Platforms of Recombinant Proteins

Many plants have been explored and used for the production of recombinant proteins and vaccine antigens (Table 2; [41, 50]). Generally, these can be divided into leafy crops, fruits and root vegetables, and seeds. Soybean, alfalfa, and corn are among the most efficient plant systems for production of foreign proteins from an economic point of view [41]. Preliminary studies to generate stably transformed transgenic plants expressing proteins of interest have often seen the use of model plants such as* Arabidopsis thaliana* and tobacco [39]. With the completion of sequencing on the* Arabidopsis* nuclear genomes [51], transgenic plant research is blossoming. A variety of* Arabidopsis* lines and mutants with accessible genetics information are available. Hence, transformation protocols of* Arabidopsis* are established and could be performed successfully even by nonspecialists. However, the plant is not useful for commercial production as it has a low biomass [50].

On the contrary, tobacco could achieve a relatively high biomass yield [52]. Besides, it is a nonfood and nonfeed crop; thus the risk of transgenic tobacco entering feed and human food chains is thus reduced [50]. However, the risk of crossing with nontransgenic tobacco in the open field production cannot be fully eliminated [41]. Moreover, transgenic tobacco cannot be consumed directly without downstream processing as it contains high amount of nicotine and other toxic alkaloids that must be removed completely before it could be delivered orally. Nevertheless, low-alkaloid varieties that require less processing are available. Besides, the suspension cultures of tobacco cells are devoid of these toxic metabolites and they can also be used to produce foreign proteins [53].

Other leafy crops that have been explored for molecular farming include alfalfa, clover, and lettuce [50]. Alfalfa and clover have a relatively established transformation protocols and they can be easily cultivated by clonal propagation [41]. The plant leaves can be consumed uncooked and this is particularly useful in the development of veterinary vaccines [50]. Moreover, these leafy crops contain a high protein level and they could achieve a large dry biomass yield per hectare of land [53]. Alfalfa could be harvested many times and up to nine times in a year. The N-glycosylation pattern in alfalfa is predominantly homogenous [50]. The consistency in the N-glycosylation process is of such importance particularly in the production of therapeutic proteins as the biological functions of these proteins are affected by the N-linked glycan structures [54, 55]. In contrast to the N-glycosylation in tobacco that showed a highly heterogeneous pattern, up to 75% of the monoclonal antibody expressed in alfalfa exhibited identical glycan structures suitable for downstream humanization processing [56]. However, they have the risk of outcrossing with nontransgenic plants in the open field production. Although having a deep root system reduces the need for chemical fertilizer, transgenic plants pose some difficulties for thorough cleaning from the production field [41].

Fruits and root vegetables like tomatoes and potatoes have also become preferred plant expression hosts. Potatoes have frequently been used because the transformation protocols to generate transgenic lines are established. Tuber extracts from the transgenic lines expressing the S1 glycoprotein gene of infectious bronchitis virus (IBV) have been shown to protect the chickens from clinical disease, as well as virus shedding upon challenge [57, 58]. Besides, microtuber production of potato is available for quick assay [41]. Foreign proteins produced are also stable and could be stored for longer periods in storage tissues without refrigeration [50]. The risk of outcrossing in the open field production is low as the plant could be clonally propagated. In addition, as the industrial processing of tuber is established, the cost of downstream processing can be greatly reduced. However, potato tuber contains a relatively low protein content [41] and it is not palatable although it can be eaten raw. While cooking can improve its palatability, it might lead to denaturation of the foreign protein, thus resulting in poor immunogenicity if it was used to produce vaccine antigens [59].

Therefore, tomatoes have become a more attractive alternative system [41], since they are palatable and can be eaten raw without cooking. Thus, vaccine antigens expressed in them do not risk to be denatured by heat treatments. The first vaccine candidate used for expression in tomatoes was the rabies virus glycoprotein [60]. Furthermore, they had been used to express the capsid proteins VP2 and VP6 of rotavirus, which were shown to be immunogenic to mice by intraperitoneal delivery [61]. The inherent high level of vitamin A in tomatoes may also help in boosting immune responses [59]. Tomatoes have a well-established industrial cultivation and processing system just like potatoes. However, the fruit are also relatively low in protein content and must be chilled after harvest in order to prevent spoilage [59]. Although freeze-drying technology is available to preserve the fruit, this adds an additional cost to the downstream processing.

Plant seeds represent another expression platform for synthesis of foreign protein and vaccine antigens, as well as their storage. In comparison to perishable plants like leafy crops and fruits, plant seeds enable long-term storage of the foreign protein produced [1]. The plant seeds are generally low in water content where most seeds have a water content of less than 10% of their total biomass, whereas in most cases the leaves contain more than 90% of water [62]. Besides, plant seeds are relatively high in protein content, which ranged from 10% to 40% of their wet weight, while in most leaves the protein percentage is less than 5% [62]. Thus, plant seeds represent a good vehicle to promote stable protein accumulation and storage. Moreover, protease activities in plant seeds are low and thus the risk of spoilage is greatly reduced as the foreign proteins produced are protected from proteolytic degradation [50]. It was demonstrated that antibodies and vaccine antigens accumulated in seeds remained stable without loss of activity for years at room temperature [63, 64]. Hence, seeds are suitable for direct consumption and useful especially for the development of animal vaccines. Industrial scale seed plantation and production are well established, beginning from cultivation, harvest, storage, distribution, and processing of the seeds [1].

Maize is the most widely used cereal crop for molecular farming [65]. The maize seeds or corn has a larger grain size and higher per hectare biomass yield compared to other cereals. The in vitro manipulation and transformation of maize have been widely studied, while its commercial production, processing, and scalability are also well established. The first commercialized corn-produced product was avidin [12] from the company ProdiGene and is available in the Sigma catalogue for diagnostic use [65]. In addition, corn has been used for the development of animal vaccines. The transmissible gastroenteritis virus (TGEV) envelope spike (S) protein expressed in corn was shown to induce protective antibodies in both piglets and gilts [66, 67]. Similarly, oral feeding of transgenic maize expressing the glycoprotein protein of NDV was shown to be immunogenic and conferred protective immunity to chicken [68].

Rice represents another leading platform for production of foreign proteins [1]. Like maize, rice has a high biomass yield, and its production, processing, and scalability have also been established. One apparent advantage of rice over maize is the reduced risk of illegitimate gene flow due to pollen release resulting from self-pollination [62]. In an oral feeding immunization trial of 2-week-old specific-pathogen-free (SPF) chickens with rice seeds expressing the VP2 protein of infectious bursal disease virus (IBDV), challenge and protection studies showed evidence of protective immunity in the chickens [69]. Barley is another commercial platform being studied other than maize and rice. The self-pollinating trait of barley is an important advantage to be considered in its development as a foreign protein production system. For example, subcutaneous injection of the F4 fimbriae adhesin protein of enterotoxigenic* E. coli* produced in barley grains was shown to induce neutralizing antibodies in mice [70].

Pea and soybean are the two commonest legume platforms being studied in plant molecular farming [1]. In one study, soybean seeds were used to express enterotoxigenic* E. coli* LT-B protein [71]. These transgenic seeds were able to induce both systemic and mucosal immunity in mice upon oral administration and conferred partial protection upon challenge. A major advantage of legumes over cereals is that the total protein content of legume grains is relatively much higher than that of cereals. Compared to total proteins (8 to 13%) from cereal grains, the total protein content in pea and soybean can be as high as 40% [72]. However, this apparent advantage is compensated by the laborious and inefficient transformation procedures of legumes in addition to their relatively lower annual grain yield and higher production cost when compared to maize and rice. Nevertheless, both pea and soybean are self-pollinating species; thus the risk of gene flow contamination is less.

Overall, one of the major disadvantages of seed-based expression is the relatively longer time-to-product period [62]. As the expression of protein is targeted to the seeds, the transgenic plants are grown through a flowering cycle to produce seeds. The assessment for the expression of foreign proteins could only begin when the seed is set. This also makes seed-based production systems become less appropriate for expression of certain foreign proteins like the influenza viral antigens [62]. Since the influenza vaccines are revised annually, the amount of seed produced in the given time may be insufficient to supply the population. Besides, seed-based production involves a flowering cycle that might increase the risk of pollen release and gene flow contamination by pollen transfer especially in the open field production system [53]. In contrast, transgenic leafy crops can be harvested before flowering and thus the risk of outcrossing is reduced.

## 5. Proof of Concept Plant-Derived Veterinary Vaccines

The tremendous developments of plant-made veterinary vaccines have been mainly due to the ability to conduct challenge experiments in specific animal species of interest [4]. Key examples on the plant-produced immunogenic proteins tested against disease challenge in target animal species are shown in Table 3. One of the first demonstrations showing protective efficacy of the plant-derived vaccine was from mink enteritis virus (MEV), where a short, linear, and neutralizing epitope from the viral VP2 capsid protein was expressed in black-eyed bean [73]. Using a plant chimeric virus particles (CVPs) approach, the short epitope was inserted into the cowpea mosaic virus and displayed on the surface of CVPs upon infection in plants. Subcutaneous injection of 1 mg of the chimeric viral particles expressing MEV peptide on the CVPs surfaces protected mink against clinical disease and challenge from the virulent MEV. In yet another study, studies showed that the VP60 protein of rabbit haemorrhagic disease virus produced from the transgenic potatoes conferred protection to rabbits against infection upon parenteral immunization [74]. It was also immunogenic and induced partial protection to the rabbits upon oral delivery of the vaccine [75].

Indeed, the most promising proof of concept for an edible plant-based animal vaccine delivered orally was against TGEV of swine [67, 79, 80]. The S protein of TGEV expressed in corn was mixed in medicated milk replacer and fed orally to 10-day-old piglets [66]. With a dose of 2 mg S protein in single feeding, the piglets were fed over a 10-day period before being challenged with a virulent TGEV orally. Compared to the control group vaccinated with the commercial vaccine, where 78% of the piglets developed diarrhoea, only 50% of the piglets fed with transgenic corn had diarrhoea. The study concluded that transgenic corn was able to confer partial protection to piglets against clinical disease and experimental challenge with virulent virus. In addition, further studies were conducted to examine vaccination with transgenic corn in gilts and the transfer of protective anti-S protein antibody to suckling piglets through colostrum [67]. In the study, all gilts were primed with a modified live TGEV vaccine orally at days 115 and 102 and intramuscularly at day 88 before farrowing. Following primary vaccination, the gilts were separated into groups and subjected to different types of booster treatments. When compared to the control group that did not receive any booster dose, gilts given a double oral booster of transgenic corn containing 26 mg of S protein at days 35 and 14 before farrowing showed a significant increase of TGEV neutralizing antibody titer in the serum, colostrum, and milk. Such responses were comparable to gilts that received modified live virus vaccine as a booster. The level of neutralizing antibody titer in milk was suggested to be adequately protective to the suckling piglets, although efficacy test was not performed in the piglets [67].

Furthermore, the possibility of conducting protective efficacy experiments in target animal has also allowed the development of plant-expressed foot-and-mouth disease virus (FMDV) vaccine for cloven-hoofed animals. The FMDV VP1 capsid protein carrying the virus neutralizing epitopes was the target of expression in various plants. Transgenic plants expressing either the complete protein or antigenic peptide of VP1 have been generated in plants like* Arabidopsis* [81], alfalfa [82, 83], and potato [84]. Earlier studies conducted in mice via intraperitoneal [81–84] and/or oral [82] delivery of the leaves extract showed the vaccine was immunogenic and protective. Moreover, the VP1 protein was also expressed with the use of plant viral display vector like the tobacco mosaic virus in tobacco leaf via the CVPs approach [85]. The entire VP1 protein expressed by the plant virus and the resulting CVPs injected intraperitoneally into mice conferred protection against viral challenge with live FMDV. Although these studies have shown an induction of protective immunity in mice, it was only later that the protective efficacy experiments was conducted in swine, one of the natural hosts for FMDV. In a related study, expression of the immunogenic VP1 peptide encompassing amino acids 128 to 164 via the CVPs approach was successfully carried out [76]. Using the bamboo mosaic virus (BaMV) as a plant viral display vector, the VP1 peptide was genetically fused to the modified coat protein gene of BaMV. Upon infection in the leaves of* Chenopodium quinoa*, the BaMV plant host, the VP1 peptide was displayed on the surface of CVPs. Two intramuscular injections with 5 mg of VP1-displaying CVPs in two-month-old SPF pigs at six weeks apart resulted in the induction of anti-FMDV neutralizing antibodies. The vaccine demonstrated a complete protection in pigs against FMDV challenge four weeks after the booster dose was administered.

In the poultry vaccine arena, several infectious pathogens of economic importance have been the attention of development of plant-made vaccines. The NDV is one of them, and the virus surface glycoprotein fusion and/or hemagglutinin neuraminidase are the targets of expression. In addition to the first approved plant-produced NDV vaccine [21] that was made in tobacco cell culture, NDV viral proteins had been expressed in other plant systems as well. Oral feeding of transgenic maize expressing the viral fusion protein was shown to be immunogenic and conferred protective immunity to chicken [68].

Besides, the IBV S1 glycoprotein contains virus neutralizing and hemagglutination-inhibiting epitopes have been the component of interest for vaccine development. By stably transforming the S1 glycoprotein gene into the potato plant, tuber extracts from the transgenic potatoes were used for vaccination and protective efficacy studies in chicken [57, 58]. Here, day-old chicks were fed orally with either 2.5 or 5 g of tuber extracts corresponding to 28.6 or 57.2 *μ*g of S1 glycoprotein and feeding was repeated at days 7 and 14. Virus challenge performed via intranasal route using the virulent IBV seven days after the final vaccination showed all chicks fed with 5 g of tuber extracts were protected from clinical disease and virus shedding. This result was comparable to the control group vaccinated with commercial modified live vaccine.

The IBDV, being a highly contagious and deadly virus of young chickens, is another important pathogen of poultry. It is a double stranded RNA virus with two genome segments, termed A and B [86]. The VP2 capsid protein of IBDV segment A contains the virus neutralizing epitopes and was selected as the component for the development of plant-made vaccine [69, 87]. In one study, the VP2 gene of the variant IBDV strain E was expressed in* Arabidopsis thaliana* [87], while in another study the VP2 gene of a virulent IBDV strain with attenuated segment A was expressed in rice seeds [69]. In the oral feeding immunization trial with rice seeds, 2-week-old SPF chickens were fed with transgenic rice seeds at weekly interval for four times before being challenged with the virulent IBDV strain [69]. Chickens fed with 5 g of transgenic rice seed containing 40.21 *μ*g of VP2 protein in a grain [69], amounting to approximately 10 mg dose of VP2 protein [88], gave the best result in challenge and protection studies. Evaluation based on lesion scoring of the bursa after challenge revealed that orally immunized chickens achieved better lesion score compared to chickens that received the live attenuated vaccine. The orally immunized chickens also contained less antigen present in the bursal tissue based on immunofluorescence assay. Furthermore, the full-length VP2 gene of a classical IBDV strain has been transiently expressed in* Nicotiana benthamiana* leaves and the recombinant protein was extracted for subunit vaccination [78]. Eighteen-day-old chicks injected intramuscularly with 12 *μ*g of VP2 protein emulsified in oil adjuvant and boosted after 22 and 35 days were shown to produce anti-IBDV antibody with neutralizing ability. Apart from VP2 protein obtained by stable or transient transformation in plants, the CVPs approach was also used to generate viable chimeric BaMV virus carrying the VP2 P domain loop P_BC_ of a very virulent IBDV [77]. Intramuscular injection with 600 *μ*g recombinant BaMV in oil adjuvant to 3-week-old SPF chickens was shown to induce IBDV-specific antibodies and protected the chickens upon challenge with a very virulent IBDV strain 28 days after vaccination. These studies concluded that plant-made VP2 protein represents a useful vaccination strategy against IBDV in chicken.

## 6. Conclusions

It is indeed surprising to see that 26 years down the road only two recombinant protein products from plants had made it through the regulatory processes to be licensed: monoclonal antibody against HBsAg and poultry vaccine against NDV. The idea of plant-made vaccines as edible vaccines has received much publicity and enthusiastic development since the first proof of concept recombinant plant-derived pharmaceutical proteins was reported. However, the progress made was not without hurdles [3]. Although the reports of successful expression of target antigens of interest were numerous, many of these failed to achieve expression levels suitable for commercialization [41]. Besides, the use of food plants for production of vaccine antigens has sprouted fears of contamination of the human food chain. Worries about regulatory issues have also deterred the development of plant-made vaccines. However, the regulatory pathway for plant molecular farming of vaccine antigens for veterinary use is far shorter when compared to products intended for human use. Therefore, this represents an opportunity that warrants the pursuit of plant-made vaccines for animals. The demonstration of safety and increase usage of plant-produced recombinant protein products in animals will lead to the acceptance and recognition of plant expression technology. This will in turn encourage their use for production of plant-based biopharmaceuticals for human use. Finally, with a better understanding of plant gene expression and molecular biology, the realisation of an ideal plant-made edible vaccine will not be far. Hence, the molecular farming of vaccines, be it for veterinary or human use, will be worth the exploration.

## Figures and Tables

**Table 1:** Various applications of plant-based expression system and key events related to their development.

Product	Plant host(s)	Importance	References
Human pharmaceutical proteins			
Human interferon-*α*	Turnip	First recombinant plant-derived pharmaceutical proteins.	[7]
Human serum albumin	Tobacco, potato	Plant-derived proteins identical to the authentic human proteins.	[9]

Antibodies			
Mouse immunoglobulin	Tobacco	First plant-derived antibody showing the ability of plants to assemble heterologous biomolecules.	[8]
Antibody against hepatitis B	Tobacco	First commercialized plant-derived antibody used for vaccine purification in Cuba.	[10]
Antibody against *Streptococcus mutans *(CaroRx)	Tobacco	EU-approved medical device for prevention of tooth decay.	[6, 11]

Industrial and agricultural recombinant enzymes			
Avidin	Maize	Plant-derived products on the market.	[12]
*β*-glucuronidase	Maize		[13]
Trypsin	Maize		[14]

Vaccine antigens- human use			
Hepatitis B antigen	Tobacco	Plant-made HBsAg particles antigenically and physically similar to HBsAg particles derived from human serum and recombinant yeast.	[15]
	Lettuce, lupin	Specific antibody responses in human volunteers fed with transgenic lettuce.	[16]
	Potato	Increased specific serum immune response when given orally as a booster to vaccinated human volunteers.	[17]
*Escherichia coli *LT-B	Potato	First proof of concept for edible plant-derived vaccines that conferred protection to mice upon oral feeding.	[18]
	Maize	Specific serum and mucosal antibody responses in human volunteers ingested transgenic corn germ meal.	[19]
Norwalk virus	Tobacco, potato	Specific serum and mucosal antibody responses in mice fed orally with transgenic plants. Phase I clinical trials completed.	[20]

Vaccine antigens- veterinary use			
Newcastle disease virus HN proteins	Tobacco suspension cell culture	First USDA-approved plant-derived veterinary vaccine.	[21]

**Table 2:** Comparison of different plant-based expression platforms for recombinant proteins production.

Plant hosts	Advantages	Disadvantages
Model plants		
*Arabidopsis thaliana *	Often used for preliminary studies. A variety of *Arabidopsis* lines and mutants with accessible genetics information are available. Small genome size and short life cycle. Self-pollinating plant that could produce numerous seeds. Exceptional ease for transformation by *Agrobacterium*-mediated approach.	Low biomass.
Tobacco	Established transformation and expression protocols. High biomass yield. Nonfood and nonfeed crop. Less risk of feed and human food chains contamination. Low-alkaloid varieties are available, which requires less processing.	Risk of crossing with nontransgenic tobacco in the open field production. Contain high nicotine and other toxic alkaloids. Direct consumption not possible.

Leafy crops		
Alfalfa Clover	Established transformation protocols. Clonal propagation is possible. Direct consumption and useful for animal vaccines. High protein level. Large dry biomass yield. Many harvests per year. Homogenous N-linked glycan structures in alfalfa.	Risk of outcrossing with nontransgenic plants in the open field production. Low protein stability. Perishable tissues and must be processed soon after harvest. Deep root system in alfalfa makes them difficult for thorough cleaning from the production field.
Lettuce	Edible raw and useful for human vaccines. Fast growing.	

Fruits and root vegetables		
Potato	Established transformation protocols. Microtuber production is available for quick assay. Stable storage for longer periods in storage tissues without refrigeration. Clonal propagation, low risk of outcrossing in the open field production. Industrial processing of tuber is well established.	Low protein content. Raw tuber is not palatable, while cooking might cause denaturation of the foreign protein.
Tomato	Palatable in raw form. High biomass yield. Inherent high level of vitamin A may help in boosting immune responses. Industrial cultivation and processing are well established.	Low protein content. Acidic in nature and may be incompatible with some antigens or use for infants. Spoil readily.

Cereal and legume seeds		
Maize	Most widely used cereal crop for molecular farming. Large grain size and high per hectare biomass yield. In vitro manipulation and transformation of maize are well studied. Commercial production, processing, and scalability are established.	Cross-pollinating plant. Concerns for contamination of food maize crops.
Rice	High biomass yield. Commercial production, processing, and scalability are established. Self-pollinating, reduced risk of illegitimate gene flow due to pollen release.	Longer time-to-product period.
Barley	Self-pollinating.	Less widely grown. Inefficient transformation system.
Pea Soybean	Higher protein content than that of the cereals. Self-pollinating are risk of gene flow contamination are less.	Laborious and inefficient transformation procedures. Lower annual grain yield and higher production cost compared to maize and rice.

**Table 3:** Summary of plant-derived immunogenic veterinary viral antigens tested against disease challenge in target animals.

Target animals	Disease antigens	Protein expressed	Plant host(s)	Expression approach	Findings	References
Mink	Mink enteritis virus	VP2 capsid protein	Black-eyed bean	CVPs using cowpea mosaic virus	Subcutaneous injection of 1 mg of the chimeric virus protected mink against clinical disease and challenge from the virulent virus.	[73]

Rabbit	Rabbit haemorrhagic disease virus	VP60 protein	Potato, leaf	Stable transformation	Animals primed subcutaneously with 12 *μ*g of recombinant protein emulsified in oil adjuvant and boosted intramuscularly after 30, 60, and 90 days were protected from challenge with the virulent virus.	[74]
			Potato, tuber	Stable transformation	Rabbits fed with 500 *μ*g of recombinant protein and boosted on 21, 42, and 63 days after primary vaccination were partially protected from challenge with the virulent virus.	[75]

Swine	Transmissible gastroenteritis virus	Envelope spike (S) protein	Maize	Stable transformation	Piglets fed with 2 mg S protein daily for 10 consecutive days prior to challenge showed fewer symptoms of infection than the control group vaccinated with commercial vaccine.	[66]
					Gilts previously primed with commercial vaccine and boosted orally two times, each with 26 mg S protein, showed a significant increase of TGEV neutralizing antibody titer in serum, colostrum, and milk.	[67]

Cloven-hoofed animals	Foot-and-mouth disease virus	VP1 capsid protein	*Chenopodium quinoa *	CVPs using bamboo mosaic virus	Two intramuscular injections with 5 mg of chimeric virus in SPF pigs at six weeks apart induced neutralizing antibodies and demonstrated a complete protection against challenge.	[76]

Poultry	Newcastle disease virus	Fusion protein	Maize	Stable transformation	Oral feeding of transgenic maize was shown to be immunogenic and conferred complete protection against challenge comparable to that by a commercial vaccine to the chicken.	[68]
	Infectious bronchitis virus	S1 glycoprotein	Potato, tuber	Stable transformation	Day-old chicks fed orally with 57.2 *μ*g of recombinant protein and boosted at 7 and 14 days after were protected from clinical disease and virus shedding upon challenge.	[57]
	Infectious bursal disease virus	VP2 capsid protein	Rice seed	Stable transformation	Oral feeding with 10 mg of recombinant protein protected chicken from challenge with a highly virulent virus, showing a better bursal lesion score compared to chickens that received the live attenuated vaccine.	[69]
			*Chenopodium quinoa *	CVPs using bamboo mosaic virus	Intramuscular injection with 600 *μ*g chimeric virus in oil adjuvant was shown to induce specific antibodies and protected SPF chickens upon challenge with a very virulent virus strain.	[77]
			*Nicotiana benthamiana *	Transient expression	Intramuscular injection with 12 *μ*g of recombinant protein emulsified in oil adjuvant with booster doses given at 22 and 35 days later was shown to produce neutralizing antibodies in chickens, with reduced T-cell infiltration into the bursa of Fabricius upon challenge.	[78]

## References

[1] Lau O. S., Sun S. S. M. (2009). Plant seeds as bioreactors for recombinant protein production. *Biotechnology Advances*.

[2] Walmsley A. M., Arntzen C. J. (2000). Plants for delivery of edible vaccines. *Current Opinion in Biotechnology*.

[3] Rybicki E. P. (2009). Plant-produced vaccines: promise and reality. *Drug Discovery Today*.

[4] Santi L. (2009). Plant derived veterinary vaccines. *Veterinary Research Communications*.

[5] Joensuu J. J., Niklander-Teeri V., Brandle J. E. (2008). Transgenic plants for animal health: plant-made vaccine antigens for animal infectious disease control. *Phytochemistry Reviews*.

[6] Spök A., Twyman R. M., Fischer R., Ma J. K. C., Sparrow P. A. C. (2008). Evolution of a regulatory framework for pharmaceuticals derived from genetically modified plants. *Trends in Biotechnology*.

[7] De Zoeten G. A., Penswick J. R., Horisberger M. A., Ahl P., Schultze M., Hohn T. (1989). The expression, localization, and effect of a human interferon in plants. *Virology*.

[8] Hiatt A., Cafferkey R., Bowdish K. (1989). Production of antibodies in transgenic plants. *Nature*.

[9] Sijmons P. C., Dekker B. M. M., Schrammeijer B., Verwoerd T. C., Van Den Elzen P. J. M., Hoekema A. (1990). Production of correctly processed human serum albumin in transgenic plants. *Nature Biotechnology*.

[10] Pujol M., Ramírez N. I., Ayala M. (2005). An integral approach towards a practical application for a plant-made monoclonal antibody in vaccine purification. *Vaccine*.

[11] Ma J. K.-C., Barros E., Bock R. (2005). Molecular farming for new drugs and vaccines. Current perspectives on the production of pharmaceuticals in transgenic plants. *EMBO Reports*.

[12] Hood E. E., Witcher D. R., Maddock S. (1997). Commercial production of avidin from transgenic maize: characterization of transformant, production, processing, extraction and purification. *Molecular Breeding*.

[13] Witcher D. R., Hood E. E., Peterson D. (1998). Commercial production of *β*-glucuronidase (GUS): a model system for the production of proteins in plants. *Molecular Breeding*.

[14] Woodard S. L., Mayor J. M., Bailey M. R. (2003). Maize (Zea mays)-derived bovine trypsin: characterization of the first large-scale, commercial protein product from transgenic plants. *Biotechnology and Applied Biochemistry*.

[15] Mason H. S., Lam D. M.-K., Arntzen C. J. (1992). Expression of hepatitis B surface antigen in transgenic plants. *Proceedings of the National Academy of Sciences of the United States of America*.

[16] Kapusta J., Modelska A., Figlerowicz M. (1999). A plant-derived edible vaccine against hepatitis B virus. *The FASEB Journal*.

[17] Thanavala Y., Mahoney M., Pal S. (2005). Immunogenicity in humans of an edible vaccine for hepatitis B. *Proceedings of the National Academy of Sciences of the United States of America*.

[18] Mason H. S., Haq T. A., Clements J. D., Arntzen C. J. (1998). Edible vaccine protects mice against *Escherichia coli* heat-labile enterotoxin (LT): potatoes expressing a synthetic LT-B gene. *Vaccine*.

[19] Tacket C. O., Pasetti M. F., Edelman R., Howard J. A., Streatfield S. (2004). Immunogenicity of recombinant LT-B delivered orally to humans in transgenic corn. *Vaccine*.

[20] Mason H. S., Ball J. M., Shi J.-J., Jiang X., Estes M. K., Arntzen C. J. (1996). Expression of Norwalk virus capsid protein in transgenic tobacco and potato and its oral immunogenicity in mice. *Proceedings of the National Academy of Sciences of the United States of America*.

[21] Vermij P., Waltz E. (2006). USDA approves the first plant-based vaccine. *Nature Biotechnology*.

[22] Ma J. K. (1999). The caries vaccine: a growing prospect. *Dental Update*.

[23] Pascual D. W. (2007). Vaccines are for dinner. *Proceedings of the National Academy of Sciences of the United States of America*.

[24] Tregoning J., Maliga P., Dougan G., Nixon P. J. (2004). New advances in the production of edible plant vaccines: chloroplast expression of a tetanus vaccine antigen, TetC. *Phytochemistry*.

[25] O'Brien G. J., Bryant C. J., Voogd C., Greenberg H. B., Gardner R. C., Bellamy A. R. (2000). Rotavirus VP6 expressed by PVX vectors in *Nicotiana benthamiana* coats PVX rods and also assembles into viruslike particles. *Virology*.

[26] Huang Z., Dry I., Webster D., Strugnell R., Wesselingh S. (2001). Plant-derived measles virus hemagglutinin protein induces neutralizing antibodies in mice. *Vaccine*.

[27] Webster D. E., Cooney M. L., Huang Z. (2002). Successful boosting of a DNA measles immunization with an oral plant-derived measles virus vaccine. *Journal of Virology*.

[28] McLain L., Durrani Z., Wisniewski L. A., Porta C., Lomonossoff G. P., Dimmock N. J. (1996). Stimulation of neutralizing antibodies to human immunodeficiency virus type 1 in three strains of mice immunized with a 22 amino acid peptide of gp41 expressed on the surface of a plant virus. *Vaccine*.

[29] Zhang G. G., Rodrigues L., Rovinski B., White K. A. (2002). Production of HIV-1 p24 protein in transgenic tobacco plants. *Applied Biochemistry and Biotechnology—Part B Molecular Biotechnology*.

[30] Meyers A., Chakauya E., Shephard E. (2008). Expression of HIV-1 antigens in plants as potential subunit vaccines. *BMC Biotechnology*.

[31] Tackaberry E. S., Dudani A. K., Prior F. (1999). Development of biopharmaceuticals in plant expression systems: cloning, expression and immunological reactivity of human cytomegalovirus glycoprotein B (UL55) in seeds of transgenic tobacco. *Vaccine*.

[32] Belanger H., Fleysh N., Shannon C. O. X. (2000). Human respiratory syncytial virus vaccine antigen produced in plants. *The FASEB Journal*.

[33] Sandhu J. S., Krasnyanski S. F., Domier L. L., Korban S. S., Osadjan M. D., Buetow D. E. (2000). Oral immunization of mice with transgenic tomato fruit expressing respiratory syncytial virus-F protein induces a systemic immune response. *Transgenic Research*.

[34] Brennan F. R., Jones T. D., Longstaff M. (1999). Immunogenicity of peptides derived from a fibronectin-binding protein of *S. aureus* expressed on two different plant viruses. *Vaccine*.

[35] Brennan F. R., Jones T. D., Gilleland L. B. (1999). Pseudomonas aeruginosa outer-membrane protein F epitopes are highly immunogenic in mice when expressed on a plant virus. *Microbiology*.

[36] Staczek J., Bendahmane M., Gilleland L. B., Beachy R. N., Gilleland H. E. (2000). Immunization with a chimeric tobacco mosaic virus containing an epitope of outer membrane protein F of *Pseudomonas aeruginosa* provides protection against challenge with *P. aeruginosa*. *Vaccine*.

[37] Turpen T. H., Reinl S. J., Charoenvit Y., Hoffman S. L., Fallarme V., Grill L. K. (1995). Malaria epitopes expressed on the surface of recombinant tobacco mosaic virus. *Nature Biotechnology*.

[38] Sala F., Rigano M. M., Barbante A., Basso B., Walmsley A. M., Castiglione S. (2003). Vaccine antigen production in transgenic plants: strategies, gene constructs and perspectives. *Vaccine*.

[39] Floss D. M., Falkenburg D., Conrad U. (2007). Production of vaccines and therapeutic antibodies for veterinary applications in transgenic plants: an overview. *Transgenic Research*.

[40] Rigano M. M., Walmsley A. M. (2005). Expression systems and developments in plant-made vaccines. *Immunology and Cell Biology*.

[41] Mason H. S., Warzecha H., Mor T., Arntzen C. J. (2002). Edible plant vaccines: applications for prophylactic and therapeutic molecular medicine. *Trends in Molecular Medicine*.

[42] Giddings G. (2001). Transgenic plants as protein factories. *Current Opinion in Biotechnology*.

[43] Mett V., Farrance C. E., Green B. J., Yusibov V. (2008). Plants as biofactories. *Biologicals*.

[44] Muskett J. C., Reed N. E., Thornton D. H. (1985). Increased virulence of an infectious bursal disease live virus vaccine after passage in chicks. *Vaccine*.

[45] Zavala G., Cheng S. (2006). Detection and characterization of avian leukosis virus in Marek's disease vaccines. *Avian Diseases*.

[46] Streatfield S. J., Howard J. A. (2003). Plant-based vaccines. *International Journal for Parasitology*.

[47] Wilson I. B. H. (2002). Glycosylation of proteins in plants and invertebrates. *Current Opinion in Structural Biology*.

[48] Brooks S. A. (2004). Appropriate glycosylation of recombinant proteins for human use. *Molecular Biotechnology*.

[49] Saint-Jore-Dupas C., Faye L., Gomord V. (2007). From planta to pharma with glycosylation in the toolbox. *Trends in Biotechnology*.

[50] Fischer R., Stoger E., Schillberg S., Christou P., Twyman R. M. (2004). Plant-based production of biopharmaceuticals. *Current Opinion in Plant Biology*.

[51] Arabidopsis Genome Initiative (2000). Analysis of the genome sequence of the flowering plant *Arabidopsis thaliana*. *Nature*.

[52] Daniell H., Streatfield S. J., Wycoff K. (2001). Medical molecular farming: production of antibodies, biopharmaceuticals and edible vaccines in plants. *Trends in Plant Science*.

[53] Twyman R. M., Stoger E., Schillberg S., Christou P., Fischer R. (2003). Molecular farming in plants: host systems and expression technology. *Trends in Biotechnology*.

[54] Lerouge P., Cabanes-Macheteau M., Rayon C., Fischette-Lainé A.-C., Gomord V., Faye L. (1998). N-glycoprotein biosynthesis in plants: recent developments and future trends. *Plant Molecular Biology*.

[55] Gomord V., Fitchette A.-C., Menu-Bouaouiche L. (2010). Plant-specific glycosylation patterns in the context of therapeutic protein production. *Plant Biotechnology Journal*.

[56] Bardor M., Loutelier-Bourhis C., Paccalet T. (2003). Monoclonal C5-1 antibody produced in transgenic alfalfa plants exhibits a *N*-glycosylation that is homogenous and suitable for glyco-engineering into human-compatible structures. *Plant Biotechnology Journal*.

[57] Zhou J.-Y., Wu J.-X., Cheng L.-Q. (2003). Expression of immunogenic S1 glycoprotein of infectious bronchitis virus in transgenic potatoes. *Journal of Virology*.

[58] Zhou J.-Y., Cheng L.-Q., Zheng X.-J. (2004). Generation of the transgenic potato expressing full-length spike protein of infectious bronchitis virus. *Journal of Biotechnology*.

[59] Gunn K. S., Singh N., Giambrone J., Wu H. (2012). Using transgenic plants as bioreactors to produce edible vaccines. *Journal of Biotech Research*.

[60] McGarvey P. B., Hammond J., Dienelt M. M. (1995). Expression of the rabies virus glycoprotein in transgenic tomatoes. *Nature Biotechnology*.

[61] Saldaña S., Guadarrama F. E., Olivera Flores T. D. J. (2006). Production of rotavirus-like particles in tomato (*Lycopersicon esculentum* L.) fruit by expression of capsid proteins VP2 and VP6 and immunological studies. *Viral Immunology*.

[62] Boothe J., Nykiforuk C., Shen Y. (2010). Seed-based expression systems for plant molecular farming. *Plant Biotechnology Journal*.

[63] Stöger E., Vaquero C., Torres E. (2000). Cereal crops as viable production and storage systems for pharmaceutical scFv antibodies. *Plant Molecular Biology*.

[64] Nochi T., Takagi H., Yuki Y. (2007). Rice-based mucosal vaccine as a global strategy for cold-chain- and needle-free vaccination. *Proceedings of the National Academy of Sciences of the United States of America*.

[65] Ramessar K., Sabalza M., Capell T., Christou P. (2008). Maize plants: an ideal production platform for effective and safe molecular pharming. *Plant Science*.

[66] Streatfield S. J., Jilka J. M., Hood E. E. (2001). Plant-based vaccines: unique advantages. *Vaccine*.

[67] Lamphear B. J., Jilka J. M., Kesl L., Welter M., Howard J. A., Streatfield S. J. (2004). A corn-based delivery system for animal vaccines: an oral transmissible gastroenteritis virus vaccine boosts lactogenic immunity in swine. *Vaccine*.

[68] Guerrero-Andrade O., Loza-Rubio E., Olivera-Flores T., Fehérvári-Bone T., Gómez-Lim M. A. (2006). Expression of the Newcastle disease virus fusion protein in transgenic maize and immunological studies. *Transgenic Research*.

[69] Wu J., Yu L., Li L., Hu J., Zhou J., Zhou X. (2007). Oral immunization with transgenic rice seeds expressing VP2 protein of infectious bursal disease virus induces protective immune responses in chickens. *Plant Biotechnology Journal*.

[70] Joensuu J. J., Kotiaho M., Teeri T. H. (2006). Glycosylated F4 (K88) fimbrial adhesin FaeG expressed in barley endosperm induces ETEC-neutralizing antibodies in mice. *Transgenic Research*.

[71] Moravec T., Schmidt M. A., Herman E. M., Woodford-Thomas T. (2007). Production of *Escherichia coli* heat labile toxin (LT) B subunit in soybean seed and analysis of its immunogenicity as an oral vaccine. *Vaccine*.

[72] Stoger E., Ma J. K.-C., Fischer R., Christou P. (2005). Sowing the seeds of success: pharmaceutical proteins from plants. *Current Opinion in Biotechnology*.

[73] Dalsgaard K., Uttentha Å., Jones T. D. (1997). Plant-derived vaccine protects target animals against a viral disease. *Nature Biotechnology*.

[74] Castañón S., Marín M. S., Martín-Alonso J. M. (1999). Immunization with potato plants expressing VP60 protein protects against rabbit hemorrhagic disease virus. *Journal of Virology*.

[75] Martín-Alonso J. M., Castañón S., Alonso P., Parra F., Ordás R. (2003). Oral immunization using tuber extracts from transgenic potato plants expressing rabbit hemorrhagic disease virus capsid protein. *Transgenic Research*.

[76] Yang C.-D., Liao J.-T., Lai C.-Y. (2007). Induction of protective immunity in swine by recombinant bamboo mosaic virus expressing foot-and-mouth disease virus epitopes. *BMC Biotechnology*.

[77] Chen T.-H., Chen T.-H., Hu C.-C. (2012). Induction of protective immunity in chickens immunized with plant-made chimeric bamboo mosaic virus particles expressing very virulent Infectious bursal disease virus antigen. *Virus Research*.

[78] Gómez E., Lucero M. S., Chimeno Zoth S., Carballeda J. M., Gravisaco M. J., Berinstein A. (2013). Transient expression of VP2 in *Nicotiana benthamiana* and its use as a plant-based vaccine against infectious bursal disease virus. *Vaccine*.

[79] Lamphear B. J., Streatfield S. J., Jilka J. M. (2002). Delivery of subunit vaccines in maize seed. *Journal of Controlled Release*.

[80] Howard J. A. (2004). Commercialization of plant-based vaccines from research and development to manufacturing.. *Animal Health Research Reviews*.

[81] Carrillo C., Wigdorovitz A., Oliveros J. C. (1998). Protective immune response to foot-and-mouth disease virus with VP1 expressed in transgenic plants. *Journal of Virology*.

[82] Wigdorovitz A., Carrillo C., Dus Santos M. J. (1999). Induction of a protective antibody response to foot and mouth disease virus in mice following oral or parenteral immunization with alfalfa transgenic plants expressing the viral structural protein VP1. *Virology*.

[83] Santos M. J. D., Wigdorovitz A., Trono K. (2002). A novel methodology to develop a foot and mouth disease virus (FMDV) peptide-based vaccine in transgenic plants. *Vaccine*.

[84] Carrillo C., Wigdorovitz A., Trono K. (2001). Induction of a virus-specific antibody response to foot and mouth disease virus using the structural protein VP1 expressed in transgenic potato plants. *Viral Immunology*.

[85] Wigdorovitz A., Pérez Filgueira D. M., Robertson N. (1999). Protection of mice against challenge with foot and mouth disease virus (FMDV) by immunization with foliar extracts from plants infected with recombinant tobacco mosaic virus expressing the FMDV structural protein VP1. *Virology*.

[86] Nick H., Cursiefen D., Becht H. (1976). Structural and growth characteristics of infectious bursal disease virus. *Journal of Virology*.

[87] Wu H., Singh N. K., Locy R. D., Scissum-Gunn K., Giambrone J. J. (2004). Expression of immunogenic VP2 protein of infectious bursal disease virus in *Arabidopsis thaliana*. *Biotechnology Letters*.

[88] Mason H. S., Herbst-Kralovetz M. M. (2012). Plant-derived antigens as mucosal vaccines. *Current Topics in Microbiology and Immunology*.

